# Ceusia-Breast: computer-aided diagnosis with contrast enhanced ultrasound image analysis for breast lesions

**DOI:** 10.1186/s12880-023-01072-9

**Published:** 2023-08-29

**Authors:** Satoshi Kondo, Megumi Satoh, Mutsumi Nishida, Ryousuke Sakano, Kazuya Takagi

**Affiliations:** 1 Muroran Institute of Technology, Hokkaido, Japan; 2 Hokkaido University Hospital, Hokkaido, Japan; 3 Konica Minolta, Inc., Tokyo, Japan

**Keywords:** Contrast-enhanced ultrasonography, Breast lesion, Computer-aided diagnosis, Support vector machines

## Abstract

**Background:**

In recent years, contrast-enhanced ultrasonography (CEUS) has been used for various applications in breast diagnosis. The superiority of CEUS over conventional B-mode imaging in the ultrasound diagnosis of the breast lesions in clinical practice has been widely confirmed. On the other hand, there have been many proposals for computer-aided diagnosis of breast lesions on B-mode ultrasound images, but few for CEUS. We propose a semi-automatic classification method based on machine learning in CEUS of breast lesions.

**Methods:**

The proposed method extracts spatial and temporal features from CEUS videos and breast tumors are classified as benign or malignant using linear support vector machines (SVM) with combination of selected optimal features. In the proposed method, tumor regions are extracted using the guidance information specified by the examiners, then morphological and texture features of tumor regions obtained from B-mode and CEUS images and TIC features obtained from CEUS video are extracted. Then, our method uses SVM classifiers to classify breast tumors as benign or malignant. During SVM training, many features are prepared, and useful features are selected. We name our proposed method "Ceucia-Breast" (Contrast Enhanced UltraSound Image Analysis for BREAST lesions).

**Results:**

The experimental results on 119 subjects show that the area under the receiver operating curve, accuracy, precision, and recall are 0.893, 0.816, 0.841 and 0.920, respectively. The classification performance is improved by our method over conventional methods using only B-mode images. In addition, we confirm that the selected features are consistent with the CEUS guidelines for breast tumor diagnosis. Furthermore, we conduct an experiment on the operator dependency of specifying guidance information and find that the intra-operator and inter-operator kappa coefficients are 1.0 and 0.798, respectively.

**Conclusion:**

The experimental results show a significant improvement in classification performance compared to conventional classification methods using only B-mode images. We also confirm that the selected features are related to the findings that are considered important in clinical practice. Furthermore, we verify the intra- and inter-examiner correlation in the guidance input for region extraction and confirm that both correlations are in strong agreement.

## Background

Contrast-enhanced ultrasonography (CEUS) uses an intravenous contrast agent to provide a detailed view of hemodynamics. CEUS can be used to diagnose lesions in various organs. Currently, the liver is the organ where CEUS diagnosis is most commonly used [1–7]. On the other hand, in Japan, the second-generation ultrasound contrast agent Sonazoid®  (perflubutane microbubbles; GE Healthcare) was approved for use in breast lesion diagnosis in 2012 [8]. In recent years, CEUS has been used for various applications in breast diagnosis, such as differentiating between benign and malignant breast lesions [9–12], examining sentinel lymph nodes [13, 14], determining the efficacy of chemotherapy [15, 16], and analyzing the correlation with pathological prognostic factors [17].

Compared with contrast-enhanced MRI and contrast-enhanced CT, CEUS has superior spatial and temporal resolution and allows the inflow and outflow of contrast agent to be observed in real time [8]. Various reports have been made on the diagnostic performance of CEUS in classifying breast lesions, and it has been shown that CEUS can achieve higher diagnostic performance than conventional ultrasonography (US), and the diagnostic performance of CEUS is almost equivalent to that of contrast-enhanced MRI [9, 10]. For example, Li et al. reported a meta-analysis comparing the diagnostic performance of US, CEUS, and a combination of US and CEUS (US+CEUS) [10]. In the comparison of US and CEUS, the sensitivity was 0.87 and 0.93, and the specificity was 0.72 and 0.86, respectively, for a total of 1545 patients and 1609 cases (751 of which were malignant) in nine studies, showing significantly better diagnostic performance of CEUS in the Area Under the Receiver Operating characteristic Curve (AUROC)^1^. When comparing US and US+CEUS, the sensitivity was 0.87 and 0.94, and the specificity was 0.80 and 0.86, respectively, for a total of 924 patients and 959 cases (505 of which were malignant) in five studies, showing that US+CEUS had significantly better diagnostic performance in AUROC. In [9], a comparison of the diagnostic performance of conventional ultrasound (B-mode and Doppler, US), CEUS, a combination of US and CEUS (US+CEUS), and MRI was compared in 61 cases (28 benign and 33 malignant). The results showed no significant difference in diagnostic performance between US and CEUS, and US+CEUS showed significantly improved diagnostic performance compared with US and CEUS. The diagnostic performance of US+CEUS was better than that of MRI, although the difference was not significant.

One of the advantages of CEUS is that it allows quantitative analysis. Time-intensity curve (TIC) analysis [18] is commonly used as the quantitative analysis method of CEUS. A TIC plots the changes in the reflective components from the contrast agent in the region of interest (ROI) against time. Curve fitting is performed on the TIC and the curve parameters are calculated and then used for quantitative analysis. A number of studies [12, 19–21] have reported the usefulness of TIC analysis in CEUS for the diagnosis of breast lesions.

Thus, the superiority of CEUS over conventional B-mode imaging in the ultrasound diagnosis of breast lesions in clinical practice has been widely confirmed. And the usefulness of TIC parameters in CEUS for quantitative analysis has also been widely confirmed. On the other hand, there have been many proposals for computer-aided diagnosis of breast lesions on B-mode ultrasound images [22–33], but few for CEUS [34].

In this study, we propose a method for semi-automatic classification of breast lesions based on machine learning and the use of CEUS. In the proposed method, tumor regions are extracted using the guidance information indicating the inside and outside of tumor regions specified by the examiners, then morphological and texture features of tumor regions obtained from B-mode and CEUS images and TIC features obtained from CEUS video are extracted. Then, our method uses SVM classifiers to classify breast tumors as benign or malignant. During the training of SVM, the features that provide maximum classification performance are selected from the morphological, texture and TIC features. Through the experiments we will show that the classification performance is significantly improved over conventional methods using only B-mode images. In addition, although the TIC is not obtained empirically for the whole tumor, but for the area where a strong contrast effect is observed in clinical practice, the proposed method automatically sets ROIs in areas where a strong contrast effect is observed, and we quantitatively confirm the usefulness of the method. Furthermore, we discuss the relationship between the features selected during training and the results obtained in clinical practice. Finally, we verify the intra- and inter-examiner dependence on the specification of the guidance information required for the analysis.

### Related works

In this section, we introduce computer-aided diagnosis techniques for breast ultrasound using B-mode images, quantitative analysis using TIC for CEUS videos, and computer-aided diagnosis techniques for breast ultrasound using CEUS videos, as related works of our proposed method.

#### Computer-aided diagnosis for breast ultrasound using B-mode images

Many computer-aided diagnostic techniques using B-mode images have been proposed for breast ultrasound. Table 1 shows a summary of representative works in this area. Table 1 arranges the references in chronological order and shows the machine learning method, the features, whether feature selection is used or not, the selected features, the number of cases used in the experiment, and the classification performance of each work. As can be seen in Table 1, SVM has been widely used until the mid-2010s. These methods use handcrafted features such as morphological and texture features, and prepare numerous features, and select useful features from them. Since the last half of the 2010s, the design of deep learning-based classification methods has been increased.

**Table 1:** Computer-aided diagnosis methods for breast ultrasound using B-mode images. Abbreviations are as followings. B: Benign, M: Malignant, Acc.: Accuracy, Sens.: Sensitivity, Spec.: Specificity, AUROC: Area Under the Receiver Operating characteristic Curve

Reference	Machine learning method	Features	Feature selection	Number of cases	Classification performance
Chang et al. 2005 [22]	SVM	6 morphological features	No	210 (B: 120, M: 90)	Acc.: 0.909, Sens.: 0.888, Spec.:0.925
Nascimento et al. 2016 [24]	Non-linear SVM	5 morphological features, 39 texture features	Yes	120 (B: 70, M: 50)	Acc.: 0.958, Sens.: 0.960, Spec.: 0.957
Wei et al. 2019 [27]	SVM	4 morphological features, 3 texture features	No	1061 (B: 472, M: 589)	Acc.: 0.873, Sens.: 0.870, Spec.: 0.876
Daoud et al. 2020 [28]	SVM	18 morphological features, 800 texture features, VGG features	Yes	643 (B: 327, M: 216)	Acc.: 0.961, Sens.: 0.957, Spec.: 0.949
Fujioka et al. 2019 [32]	CNN (GoogLeNet [35])	–	–	360 (B: 144, M: 216)	Acc.: 0.925, Sens.: 0.958, Spec.: 0.875, AUROC: 0.913
Lazo et al. 2020 [31]	CNN (VGG-16 [36])	–	–	947 (B: 587, M: 360)	Acc.: 0.919, AUROC: 0.934
Luo et al. 2023 [33]	Spatial attention CNN (for images) MLP (for BIRADS descriptors)	25 BIRADS descriptors (Manually annotated)	–	596 (B: 291, M: 305)	Acc.: 0.910, Sens.: 0.928, Spec.: 0.890, AUROC: 0.945

#### Quantitative analysis using TIC for CEUS videos

In Table 2, we show representative works on quantitative analysis methods using TIC in breast CEUS. These works do not use machine learning methods, but rather statistical tests to determine whether individual TIC parameters are significantly different between benign and malignant cases, and to obtain statistical discriminative power using a single TIC parameter.

**Table 2:** Quantitative analysis method for breast ultrasound using TIC obtained from CEUS videos. Abbreviations are as followings. TTP: Time To Peak, PI: Peak Intensity, WIS: Wash-In Slope, MTT: Mean Transit Time, AUTIC: Area Under the Time Intensity Curve, B: Benign, M: Malignant, AUROC: Area Under the Receiver Operating characteristic Curve

Reference	TIC parameters	Significant TIC parameters	Number of cases	Classification performance
Nemcova et al. 2015 [19]	TTP, PI, WIS	WIS, TTP	120 (B: 67, M: 43)	AUROC (with WIS): 0.735 AUROC (with TTP): 0.697
Zhao et al. 2017 [20]	PI, TTP, WIS, MTT	PI, Relative PI, Relative AUTIC, Relative start time	304 (B: 161, M: 143)	AUROC (with Relative PI): 0.919
Janu et al. 2020 [12]	TTP, PI, WIS, AUTIC	TTP, WIS	213 (B: 146, M: 67)	AUROC (with TTP): 0.678 AUROC (with WIS): 0.698

#### Computer-aided diagnosis for breast ultrasound using CEUS videos

We found only one paper [34] on computer-aided diagnosis for breast CEUS. In [34], a B-mode image and a CEUS video are used as input, and the features obtained by processing the image and the video with separate deep neural networks are integrated to classify them as benign or malignant. The evaluation is performed with 10-fold cross-validation using 268 cases (122 benign, 146 malignant). The results are 0.902 accuracy, 0.914 recall, 0.952 precision, and 0.932 F1 score 2.

### Contribution of our proposed method

The proposed method in [12] shows a very high performance. On the other hand, the classification performance cannot be guaranteed when the amount of data is limited, and the explainability of the classification results is low because the method is based on deep learning. The latter issue is particularly important from the perspective of real-world clinical use. There is a study [37] on improving the explainability of a deep learning model by visualizing regions of interest using GradCAM [38], but visualizing regions of interest alone does not provide a high level of explainability with respect to clinical use.

In addition, it has been empirically used in clinical settings to obtain TIC for CEUS not over the entire tumor region, but over a small region of high intensity in the tumor region [19] (hereafter referred to as "hot spot"). However, no study has verified whether it is more useful to use TIC measured over the whole tumor or TIC measured over hot spots.

In this paper, we propose a computer-aided diagnosis technique for breast CEUS based on machine learning. In the proposed method, morphological and texture features are extracted from both B-mode and CEUS images, and TIC from CEUS images, and then the case is classified into benign and malignant using linear SVM. The proposed method prepares numerous features, and selects useful features from them during the training of SVM. We show that the selected features in our proposed method are consistent with the features considered important in clinical practice. It can be said that the explainability of the classification results can be ensured, for example, by presenting a typical distribution and a value for each feature. In addition, we investigate the usefulness of obtaining a TIC for a hot spot rather than the entire tumor. Since our method requires the examiners to manually specify some guidance information in order to extract tumor regions, we will also conduct experiments to investigate the dependency of the guidance information specification.

## Materials and methods

### Overview of Ceucia-Breast

**Fig. 1:**
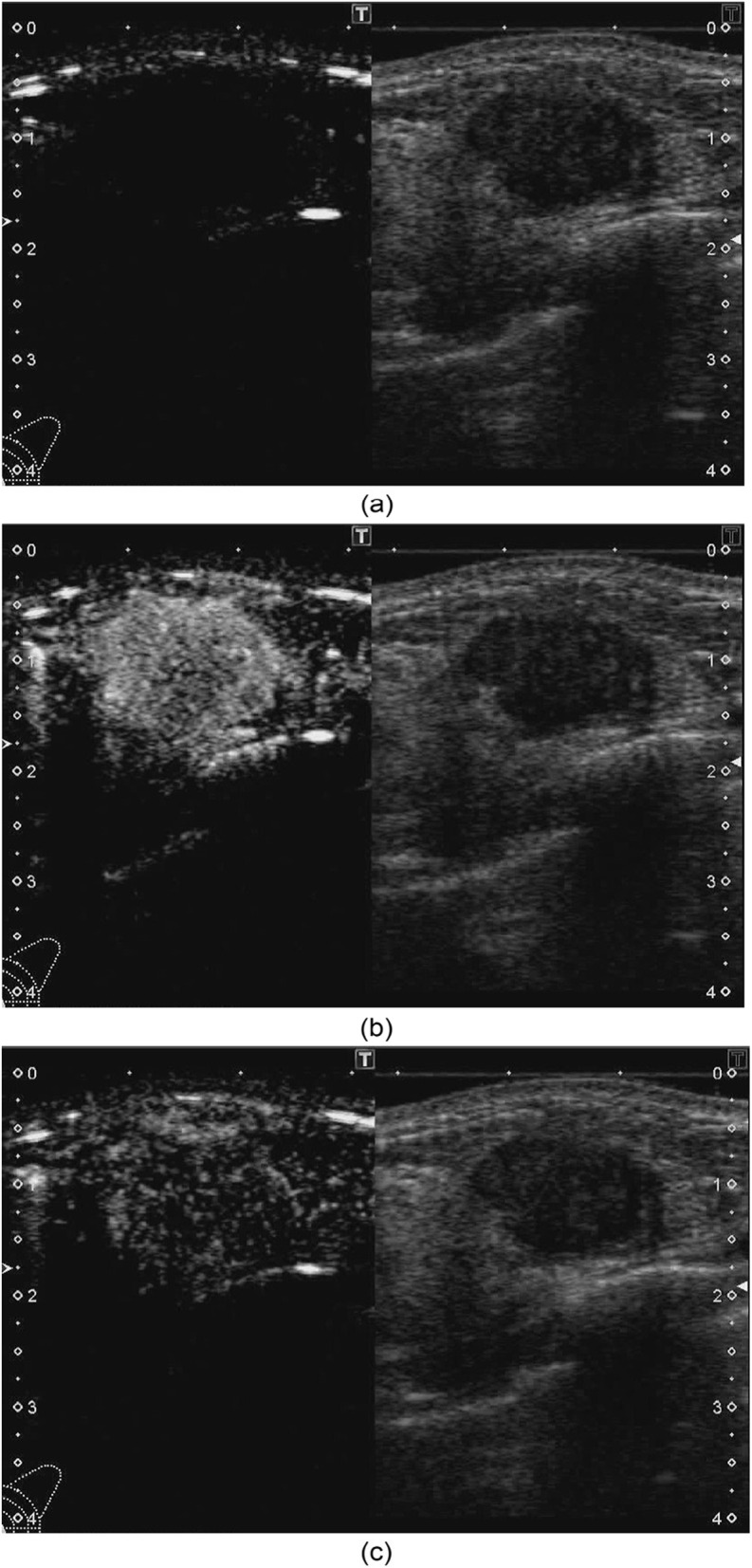
Examples of images in CEUS. The left side is the contrast image, and the right side is the B-mode image. **a** At contrast injection. **b** 20 seconds after contrast injection (in arterial phase). **c** 30 seconds after contrast injection (in venous phase). The phases are defined in [39]

With the proposed method, we recorded a breast CEUS video using an ultrasound diagnostic machine and then analyzed the recorded video. Figure 1 shows examples of the recorded images. The images and videos recorded by the ultrasound diagnostic machine are arranged with non-enhanced images on the right (hereafter, we refer to this image as “B-mode image”^3^) and the contrast-enhanced images on the left (hereinafter, we refer to this image as “contrast image”). The B-mode and contrast images in the same image correspond to the same location and time.

The proposed method uses morphological and texture features obtained from the B-mode and contrast images, region overlap features that indicate the degree of overlap of tumor regions in the B-mode and contrast images, and TIC-based features that indicate the temporal change in contrast intensity of the tumor region or hot spot. These features are described in the following sections.

### Features used in proposed method

Table 3 shows the list of features used in our proposed method. As morphological features, we use 16 features proposed in [23]. As texture features, 22 features obtained from the gray level co-occurrence matrix (GLCM) are used [24, 40, 41]. Two region overlap features, Jaccard and Dice, are obtained as features indicating the overlap of tumor regions in the B-mode and contrast images. For details on how to compute these features, please refer to [23, 40]

**Table 3:** List of features used in our proposed method

Features (Number of features)	BUS	CEUS	Features
Morphological features (16)	$\checkmark$	$\checkmark$	Perimeter, Area, Enc, LS ratio, Aspect ratio, Form factor, Roundness, Solidity, Convexity, Extent, TCA ratio, TEP ratio, TEP diff, TCP ratio, TCP diff, AP ratio
Texture features (22)	$\checkmark$	$\checkmark$	Autocorrelation, Correlation, Correlation-I, Correlation-II, Cluster Prominence, Cluster shade, Dissimilarity, Energy, Entropy, Homogeneity-I, Homogeneity-II, Maximum probability, Sum of squares:Variance, Sum average, Sum entropy, Sum variance, Difference variance, Difference entropy, Information measure of correlation 1, Information measure of correlation 2, Inverse difference normalized, Inverse difference moment normalized
Region overlap (2)	$\checkmark$		Jaccard, Dice
TIC (8)	–	$\checkmark$	Peak intensity, Time-to-peak, AUTIC-all, AUTIC-in, AUTIC-out, Wash-in slope, Wash-out slope, Full width at half maximum

**Fig. 2:**
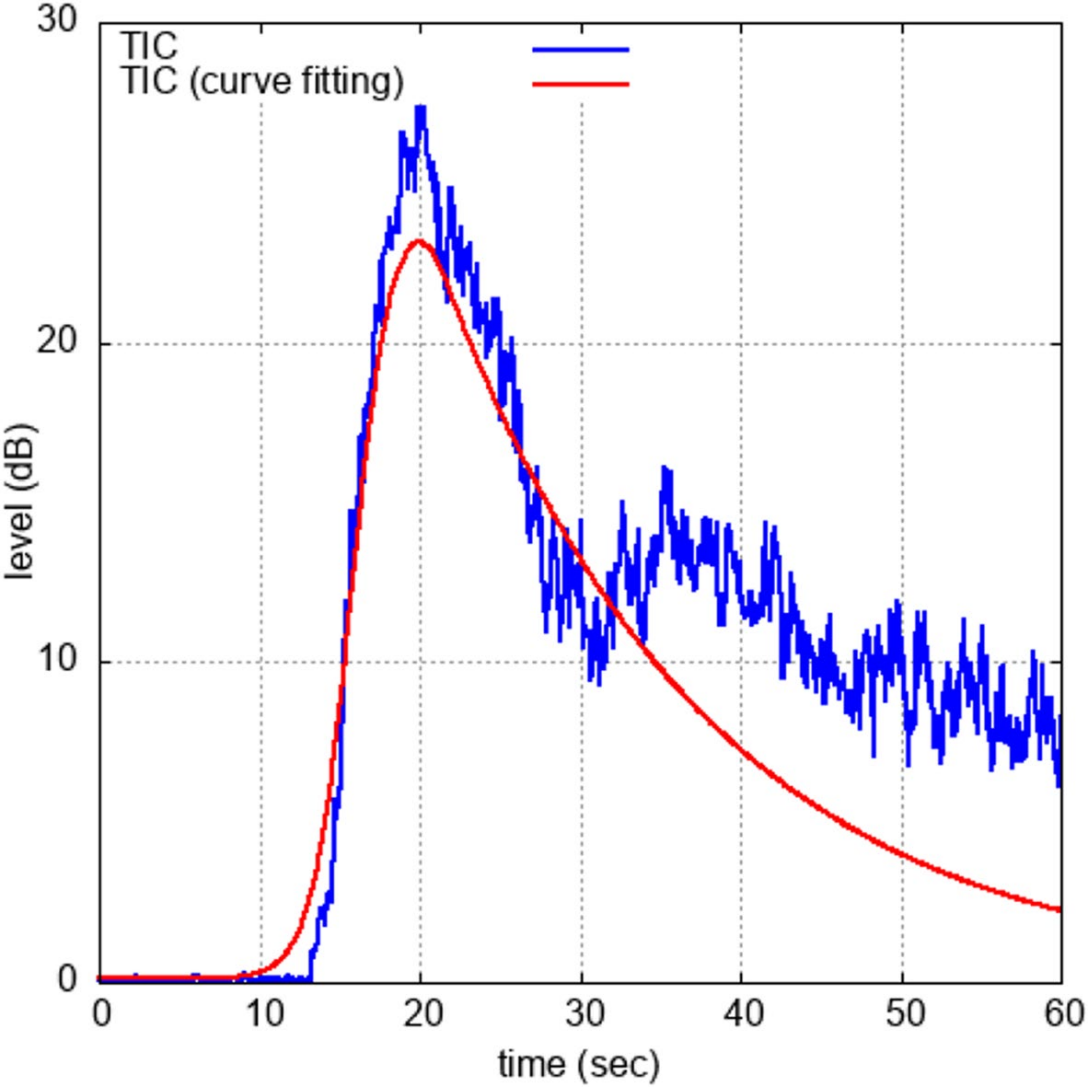
An example of TIC. The blue and red lines show the TICs before and after the curve fitting, respectively

The contrast agent is administered intravenously and flows into the mammary gland within approximately 20 seconds after intravenous injection. The contrast agent then circulates throughout the body and is primarily expelled with the breath. Therefore, TIC increases rapidly until approximately 20 seconds after administration and then gradually decreases. Equation (1) models the characteristics of TIC. In our proposed method, TIC is fitted to a curve. Equation (1) is used as the model function *f*(*t*) to fit the curve and we have eight types of TIC features.

1 $$\begin{aligned} f(t) = \left\{ \begin{array}{ll} \frac{a_{1}}{1 + \exp \{-a_{2} \times ( t - a_{3} ) \}} + a_{4} &{} ; 0 \le t \le t_{p} \\ a_{1} \times \exp (-a_{5} \times (t^{a_{6}})) &{} ; t_{p}< t < T \end{array} \right. \end{aligned}$$,

where *t* is time, $a_{i} (i = 1, 2, \cdots , 6)$ are the curve parameters, $t_{p}$ is the time at which the TIC reaches its maximum value, and *T* is the TIC length. Conceptually, $a_{1}$ is equivalent to the peak value of the TIC, $a_{2}$ is the rate at which the curve rises, $a_{3}$ is the time offset, $a_{4}$ is the offset of the contrast intensity axis, and $a_{5}$ and $a_{6}$ are the rate at which the curve falls. An example of the TIC before and after curve fitting is shown in Fig. 2.

Eight features, shown in Table 3, are obtained from the TICs and the curve fitting parameters. Peak intensity is the maximum value in the TIC, and it is equivalent to $a_{1}$ in (1). Time-to-peak is the time required to reach the peak intensity and is equal to $t_{p}$ in (1). AUTIC-all, AUTIC-in and AUTIC-out are the Area Under the Time Intensity Curves (AUTIC)^4^. AUTIC represents the area between the x-axis and the TIC. AUTIC is obtained with (2).

2 $$\begin{aligned} \text{ AUTIC } = \int _{t_{1}}^{t_{2}} f(t) dt, \end{aligned}$$

and the AUTIC-all feature is the AUTIC value for the whole TIC, the AUTIC-in feature is the AUTIC value from time 0 to $t_{p}$, the AUTIC-out feature is the AUTIC value from time $t_{p}$ to *T*. The wash-in slope feature is the rate of change of intensity from the rise of TIC to the peak value, and is the value obtained by dividing the peak intensity by $(t_{p} - a_{3})$. The wash-out slope feature is the rate of change of intensity after time $t_{p}$. Finally, the full width at half maximum feature is the time from when the TIC exceeds half the peak intensity to when it falls below half the peak intensity.

### Procedure to extract features

**Fig. 3:**
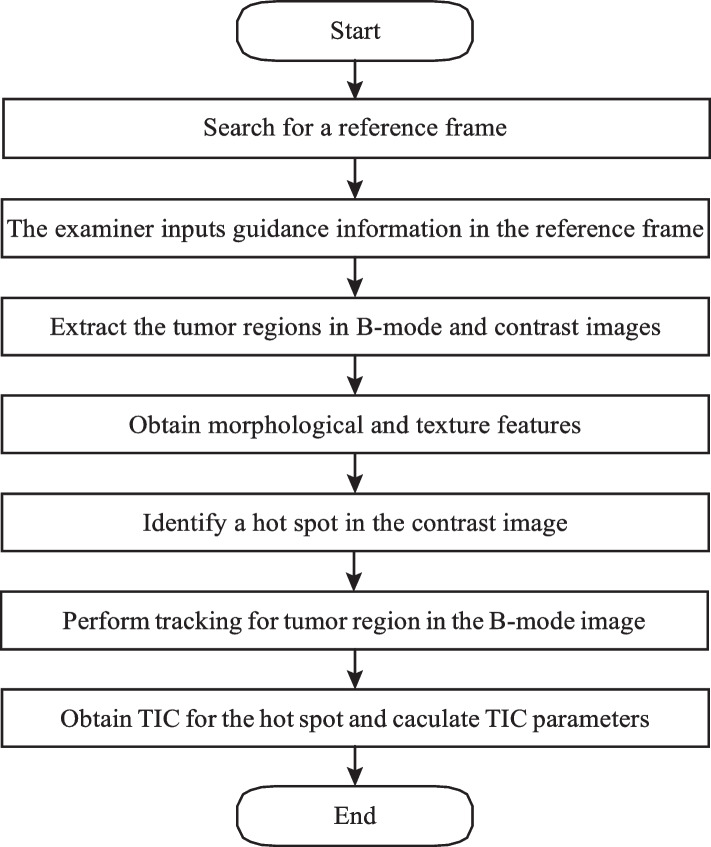
A flowchart to extract the features from CEUS video

The feature extraction procedure in the proposed method is illustrated in Fig. 3. The first step is to search for a reference frame in the input video. A reference frame is a frame for which the examiner specifies guidance information for extracting the tumor region. Although it is desirable to select a reference frame that has the maximum average intensity value of the tumor region in the contrast image, the tumor region cannot be known before the guidance information is specified. Therefore, in our proposed method, the frame with the largest average intensity value in the contrast image, i.e., averaged not the tumor region but over the whole image, is used as the reference frame. In several cases, we checked the average intensity value of the tumor area and the frames selected with the average intensity in the whole image, but all of them differed by a few frames, and we judged that there was no problem. Before the feature extraction process, the pixel values of the B-mode and contrast images are multiplied by the dynamic range obtained from the ultrasound diagnostic machine to standardize the level criteria between cases.

**Fig. 4:**
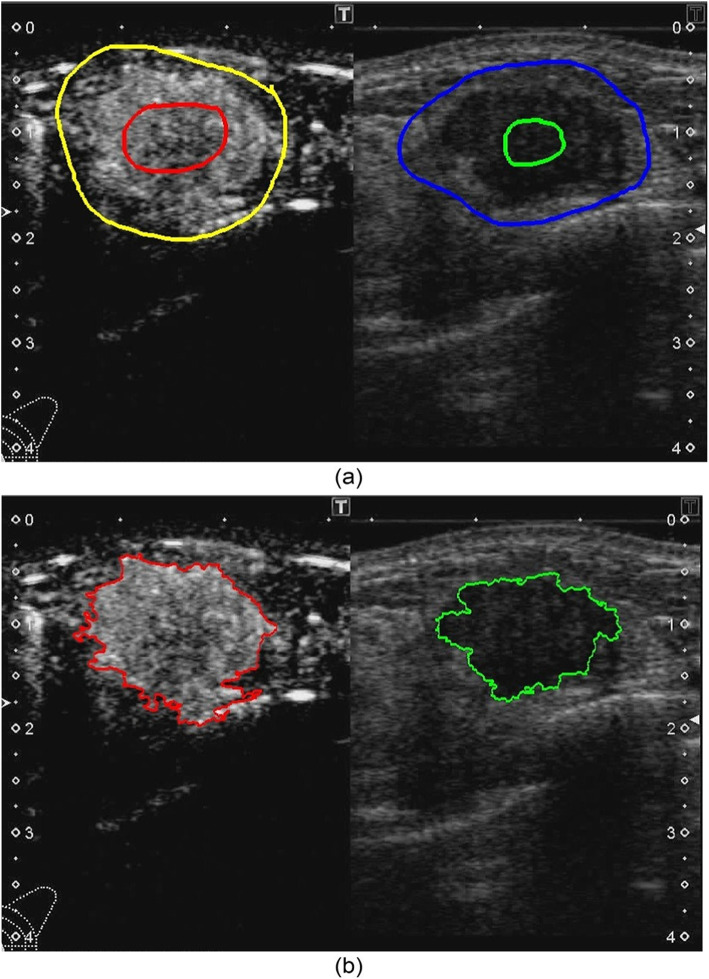
An example of tumor region extraction. **a** Guidance information. The red (inner) and yellow (outer) lines show inside and outside the tumor area in the contrast image (left side), respectively. The green (inner) and blue (outer) lines show inside and outside the tumor area in the B-mode image (right side), respectively. **b** Results of tumor region extraction. The red (left) and green (right) lines show the contours of tumor regions in the contrast and B-mode images, respectively

In the reference frame, the examiner inputs guidance information indicating the inside and outside of the tumor, respectively. The proposed method uses this guidance information to extract the tumor region on the B-mode and contrast images, respectively, using GrabCut [42]. Figure 4 shows examples of the guidance information and the extracted tumor regions. It is worth noting that the guidance information is the only one that requires manual input by the examiner in the proposed method.

For each of the extracted tumor regions in the B-mode and contrast images, 16 morphological features and 22 texture features as described in “Features used in proposed method” subsection are obtained. After extracting the tumor regions in the B-mode and contrast images, two region overlap features described in “Features used in proposed method” subsection are obtained.

The next step is to automatically extract the most hyper-enhanced region, or hot spot, from the tumor area in the contrast image. The size of the hot spot should be 2 to 5 mm square. The hot spot can be detected using the sliding window technique. A TIC is calculated for the detected hot spot area. The proposed method obtains the TIC for 60 seconds of CEUS video. Since the position of the tumor in the image changes in each frame due to slight displacements of the probe or the patient’s breathing and heartbeat, the tumor area is tracked by a tracking technique. The tracking is performed using the B-mode image because the intensity of the B-mode image changes very little, but the intensity of the contrast image changes significantly when the contrast agent reaches the breast, as shown in Fig. 1. The template used for tracking is an image of a rectangular region surrounding the tumor region in the B-mode image in the reference frame. For the remaining frames, the translational shift relative to the reference frame is obtained using a Kernelized Correlation Filter [43]. The eight TIC features described in the “Features used in proposed method” subsection are computed from the obtained TIC.

The above process results in a total of 86 features: 38 features for the B-mode image, 46 features for the contrast image, and two region overlap features. As a preprocessing step for classification, standardization is performed for each feature using the mean and standard deviation of the training data.

### Lesion classification

In the proposed method, linear SVM is used to classify the breast lesions as benign or malignant. When training the classifier, instead of using all the features described in the “Features used in proposed method” subsection, the best combination of features is selected as follows.

In feature selection during training, the importance of features is first determined by the Recursive Feature Elimination method [44] using training data. Then, the feature with the highest importance is selected, and the classification performance of the selected feature is evaluated by the leave-one-out method with the training data. Then, the classification performance with the features up to the second most important among the features is evaluated by the leave-one-out method on the training data. This process of evaluating the classification performance by increasing the number of features one by one in order of importance is repeated until all the features are selected. Then, the feature combination with the highest AUROC is selected. Finally, the obtained feature combinations are used to evaluate the performance of the classifier. We explain the method to evaluate the classification performance depending on the experiments.

## Results and discussion

### Dataset

The study includes 119 cases of 119 patients who were examined and underwent CEUS at Hokkaido University Hospital between August 2015 and December 2016. All subjects were female with a mean age of $56.6 \pm 13.6$ years old.

The Ethical Review Board of Hokkaido University Hospital approved this study. The number of the approved research study is 015-0147. Informed consent was obtained from all patients. The following breast lesions were studied: 33 benign and 86 malignant. Malignant tumor diagnoses were confirmed by biopsy or a histopathologic examination after surgery. Benign tumor diagnoses were confirmed by comprehensive clinical imaging studies using CT or MRI for at least 1 year. These diagnostic results were used as ground truth in our experiments. The mean diameter of the breast tumors was $19.1 \pm 14.1$ mm. The ultrasound machine used in the experiments was Aplio 500 (Canon Medical Systems Corporation, Otawara, Japan). The probe was PLT-1005BT. The contrast agent Sonazoid®  0.015ml/kg/body was rapidly infused and flushed with 10-15 ml of physiological saline solution using a 22G Suflo infusion needle. The guidance information for extracting tumor regions was specified by the Japan Society of Ultrasonics in Medicine (JSUM) Registered Medical Sonographers in surface anatomy.

In the following, we first discuss the results of verifying how the difference in hot spot size affects the classification performance of the proposed method. Next, we present the comparison results between the proposed method and conventional methods. Finally, the results of the verification of the examiner dependence on the guide specification are discussed.

### Verification of hot spot size

In this section, we describe the verification results of how the hot spot size affects the classification performance in the proposed method. The evaluation metrics in this experiment are AUROC, accuracy, precision (positive predictive value), recall (sensitivity), F1 score, specificity, and negative predictive value. In total, we used 119 cases, and the metrics and their 95 % confidence intervals were obtained using the Bootstrap method [45] after feature selection was performed using the method described in the “Lesion classification” section. We evaluated the proposed methods in two cases; the first using 48 features obtained from contrast images and feature selection (referred to as “CEUS”), and the second using a total of 86 features for B-mode and contrast images with feature selection (referred to as “BUS+CEUS”). Comparisons were made for hot spot sizes ranging from 2 to 5 mm, as well as for the case where the TIC is obtained over the entire tumor area, i.e., without a hot spot. Since the cases used in the experiments were imbalanced between benign and malignant, weights proportional to the inverse of the class frequency were used in the SVM training.

Tables 4 and 5 show the experimental results. These tables show the number of features using feature selection and their respective metrics for each hot spot size. Table 4 shows the results for “CEUS” and Table 5 shows the results for “BUS+CEUS”. The results in Table 4 show that although the hot spot size that shows the best performance varies depending on the evaluation metrics, the classification performance with a hot spot size of 2 mm is the highest in AUROC. Table 5 shows that the classification performance for most evaluation metrics is higher when the hot spot size is set to 2 mm than when the TIC is calculated for the whole tumor. This confirms that the TIC analysis for hot spots, which has been used in clinical practice as qualitatively useful [19], is also quantitatively useful.

**Table 4:** Comparison of classification performance by hot spot size in “CEUS”. All units except AUROC are in percent. Values in parentheses are 95 % confidence interval

Hot spot size (mm)	2	3	4	5	Entire tumor
Number of selected features	5	4	4	4	16
AUROC	**0.862** (0.792 - 0.924)	0.855 (0.780 - 0.922)	0.847 (0.776 - 0.912)	0.851 (0.776 - 0.919)	0.856 (0.778 - 0.921)
ACC	0.788 (0.717 - 0.850)	0.795 (0.733 - 0.850)	0.790 (0.733 - 0.850)	0.796 (0.733 - 0.850)	**0.797** (0.733 - 0.850)
Precision (PPV)	0.825 (0.754 - 0.902)	**0.832** (0.759 - 0.895)	0.824 (0.750 - 0.889)	0.831 (0.760 - 0.889)	0.825 (0.759 - 0.889)
Recall (Sensitivity)	0.898 (0.814 - 1.000)	0.897 (0.791 - 1.000)	0.903 (0.814 - 1.000)	0.902 (0.814 - 1.000)	**0.913** (0.814 - 1.000)
F1	0.858 (0.817 - 0.899)	0.862 (0.817 - 0.901)	0.860 (0.818 - 0.897)	0.863 (0.815 - 0.903)	**0.865** (0.824 - 0.905)
Specificity	0.510 (0.176 - 0.765)	**0.535** (0.235 - 0.765)	0.502 (0.176 - 0.765)	0.527 (0.235 - 0.765)	0.504 (0.235 - 0.706)
NPV	0.680 (0.500 - 1.000)	0.692 (0.529 - 1.000)	0.693 (0.526 - 1.000)	0.703 (0.529 - 1.000)	**0.712** (0.545 - 1.000)

Bold numbers indicate the best hot spot size within each metric

**Table 5:** Comparison of classification performance by hot spot size in “BUS+CEUS”. All units except AUROC are in percent. Values in parentheses are 95 % confidence interval

Hot spot size (mm)	2	3	4	5	Entire tumor
Number of selected features	7	13	12	12	13
AUROC	**0.875** (0.795 - 0.936)	0.865 (0.795 - 0.926)	0.862 (0.791 - 0.928)	0.865 (0.791 - 0.934)	0.868 (0.791 - 0.934)
ACC	**0.816** (0.750 - 0.883)	0.805 (0.750 - 0.867)	0.803 (0.750 - 0.867)	0.814 (0.750 - 0.867)	0.809 (0.750 - 0.867)
Precision (PPV)	0.841 (0.764 - 0.905)	0.838 (0.765 - 0.900)	0.834 (0.764 - 0.897)	**0.842** (0.769 - 0.905)	0.835 (0.774 - 0.889)
Recall (Sensitivity)	**0.920** (0.814 - 1.000)	0.907 (0.814 - 1.000)	0.909 (0.814 - 1.000)	0.915 (0.814 - 1.000)	0.918 (0.814 - 1.000)
F1	**0.877** (0.828 - 0.921)	0.869 (0.822 - 0.913)	0.869 (0.824 - 0.911)	0.876 (0.825 - 0.913)	0.873 (0.828 - 0.913)
Specificity	0.550 (0.235 - 0.765)	0.548 (0.235 - 0.765)	0.536 (0.235 - 0.765)	**0.558** (0.294 - 0.765)	0.535 (0.294 - 0.706)
NPV	**0.749** (0.562 - 1.000)	0.718 (0.550 - 1.000)	0.720 (0.555 - 1.000)	0.740 (0.556 - 1.000)	0.737 (0.562 - 1.000)

Bold numbers indicate the best hot spot size within each metric

### Comparison with conventional methods

This section describes the experimental results of comparing the performance of the proposed method with that of the conventional methods. We use classification methods for breast ultrasound using only B-mode images as the conventional methods. It is not possible to directly compare the experimental results of the proposed method presented in the “Verification of hot spot size” section with those of the previous works presented in the “Related works” section. For this purpose, we use the classification results using 38 features obtained from the B-mode images with feature selection as a conventional method. This method will be referred to as “BUS” in the following.

The evaluation metrics in this experiment are AUROC, accuracy, precision (positive predictive value), recall (sensitivity), F1 score, specificity, and negative predictive value. We use 119 cases, and the metrics and their 95 % confidence intervals were obtained using the Bootstrap method [45] after feature selection was performed using the method described in the “Lesion classification” section. The proposed method was evaluated in two cases; one using 48 features obtained from contrast images and feature selection (referred to as “CEUS”), and the other using a total of 86 features for the B-mode and contrast images (referred to as “BUS+CEUS”). From the results shown in the “Verification of hot spot size” section, we used a 2 mm hot spot size in our proposed method. As in the experiments in the “Verification of hot spot size” section, weights proportional to the inverse of the class frequency were used in the SVM training.

Table 6 shows the experimental results. The number of features selected by the feature selection and their respective metrics are shown in Table 6, as in Tables 4 and 5. Table 6 shows that the proposed method (BUS+CEUS) can significantly improve the classification performance in most of the evaluation metrics compared to the conventional method (BUS).

**Table 6:** Comparison of conventional method (BUS) and proposed methods (CEUS, BUS+CEUS). All units except AUROC are in percent. Values in parentheses are 95 % confidence interval

Method	Conventional (BUS)	Ours (CEUS)	Ours (BUS+CEUS)
Number of selected features	5	5	7
AUROC	0.742 (0.642 - 0.833)	0.862 (0.792 - 0.924)	**0.875** (0.795 - 0.936)
ACC	0.718 (0.650 - 0.767)	0.788 (0.717 - 0.850)	**0.816** (0.750 - 0.883)
Precision (PPV)	0.737 (0.712 - 0.789)	0.825 (0.754 - 0.902)	**0.841** (0.764 - 0.905)
Recall (Sensitivity)	**0.947** (0.791 - 1.000)	0.898 (0.814 - 1.000)	0.920 (0.814 - 1.000)
F1	0.827 (0.771 - 0.860)	0.858 (0.817 - 0.899)	**0.877** (0.828 - 0.921)
Specificity	0.140 (0.000 - 0.412)	0.510 (0.176 - 0.765)	**0.550** (0.235 - 0.765)
NPV	0.402 (0.000 - 1.000)	0.680 (0.500 - 1.000)	**0.749** (0.562 - 1.000)

Bold numbers indicate the best method within each metric

In this experiment, Table 7 shows the features selected in each method. These features are listed in the order of importance selected by the Recursive Feature Elimination method.

**Table 7:** Selected features. “(B)” and “(C)” means the feature is selected from the B-mode and contrast image, respectively

Method	Selected features
BUS	TEP diff(B), Info measure correlation 2(B), Aspect ratio(B), Perimeter(B), Extent(B)
CEUS	Wash-in slope, Convexity(C), Inverse difference moment normalized(C), Dice, Jaccard
BUS+CEUS	Wash-in slope, Convexity(C), Inverse difference moment normalized(C), Dice, Aspect ratio(B), Extent(B), Cluster shade(C)

**Fig. 5:**
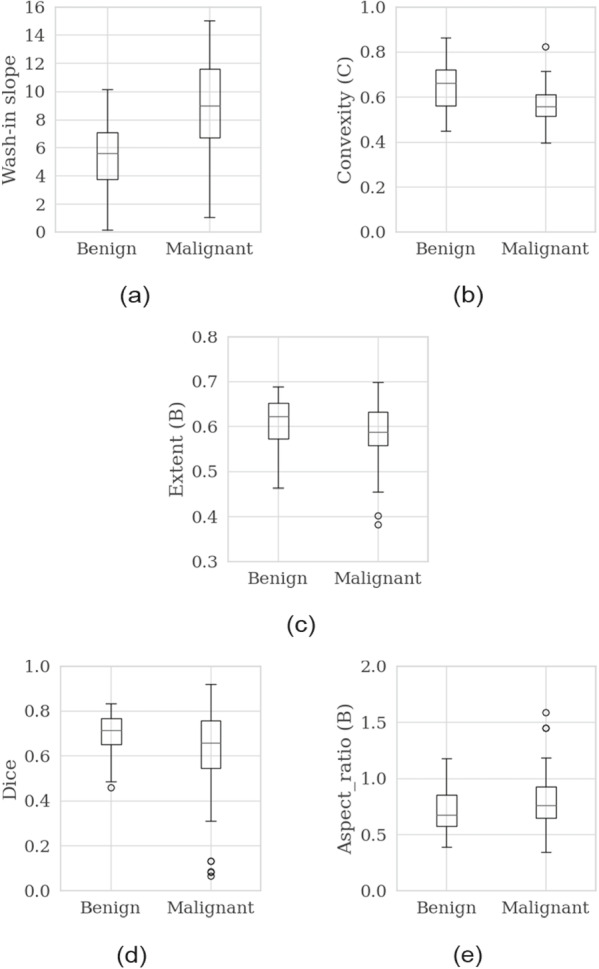
Box-and-wicker diagrams of features selected in “BUS+CEUS”. **a** Wash-in slope, (**b**) Convexity in contrast image, (**c**) Extent in B-mode image, (**d**) Dice, (**e**) Aspect ratio in B-mode image

We discuss here the features selected in BUS+CEUS. Figure 5(a) shows the distribution of the most important feature, the wash-in slope of TIC, in benign and malignant cases as a box-and-whisker plot. As can be seen in Fig. 5(a), the wash-in slope is higher for malignant cases than in benign cases. This is consistent with the characterization “Malignant lesions show early wash-in with more intense enhancement and fast wash-out compared to benign masses” reported in a survey paper [46].

Figure 5(b) shows the distribution of the second important feature, convexity, as a morphological feature of the tumor region in the contrast image. Convexity is the perimeter of the convex hull of the tumor region divided by the perimeter of the tumor region. The more convex the tumor region, the larger the value, close to 1. As can be seen in Fig. 5(b), the convexity in the contrast image shows lower values for malignant than benign tumors in our experimental results. Extent, a morphological feature of the tumor region, in the B-mode image. Extent is the tumor area divided by the area of the rectangle circumscribed by the tumor and takes a large value for areas with smooth boundaries and a small value for areas with irregular boundaries. As shown in Fig. 5(c), in our experimental results, extent has lower values for malignant tumors than for benign tumors. A guideline for breast ultrasound diagnosis [47] states that “localized masses with clear boundaries are benign, while irregular masses with speculated boundaries are often malignant,’, and the experimental results shown in Fig. 5(b) and (c) are consistent with this finding.

In addition, the distribution of the Dice feature is shown in Fig. 5(d) as a box-and-whisker plot. Dice is a measure of the overlap between a tumor region in the B-mode image and that in the contrast image, and takes 0 if there is no overlap and 1 if there is complete overlap. The guideline [47] states that “malignant tumors have more contrast than B-mode”, and the result shown in Fig. 5(d) is consistent with this finding.

Finally, the aspect ratio, a morphological feature of the tumor area, in the B-mode image is the height of the tumor divided by its width, and is greater than 1 for a vertical shape and less than 1 for a horizontal shape. The guideline [47] states that “malignant tumors are generally larger than benign tumors, and the standard value for distinguishing between benign and malignant tumors is 0.7, but we should be aware that both benign and malignant tumors gradually decrease as the tumor diameter increases.” The aspect ratio distribution for the B-mode image shown in Fig. 5(e) is consistent with this finding.

In this way, most, but not all, of the selected features in our proposed method are consistent with the features considered important in clinical setting. Therefore, when the proposed method is applied in clinical sites, the explainability of the classification results can be ensured, for example, by presenting a typical distribution and a value for each feature.

### Examiner dependency

The examiner specifies the guidance information, which is the only information that requires manual input, in our proposed method for extracting tumor regions, as described in the “Procedure to extract features” section. Therefore, the guidance information provided by different examiners may lead to different classification results. Also, the same examiner may give different guidance information over time, which may lead to different classification results.

In this section, we describe the results of an experiment to verify the effect of guidance information on classification performance. To validate the examiner dependency, we divided 119 cases into two groups: Group 1 and Group 2. Group 1 consists of 99 cases (23 benign and 76 malignant), and Group 2 consists of the remaining 20 cases (10 benign and 10 malignant). A random split of the data was performed, with the number of cases in each group fixed. In this experiment, two examiners (examiner A and examiner B, who are JSUM Registered Medical Sonographers) independently entered the guidance information. Examiner A entered guidance information for all 119 cases, and four weeks later entered guidance information for the cases in Group 2. Examiner B entered guidance information only once for the cases in Group 2. A classifier (SVM) was trained on the Group 1 cases, with the guidance information provided by examiner A, and the classification performance for the Group 2 cases was evaluated. The features used in the classifier were combinations of seven features described in the “Verification of hot spot size” section.

In this way, we obtained three sets of classification results: the first one is for the first input of guidance information by examiner A; the second one is for the second input of guidance information of examiner A; and the third one is for the input of guidance information by examiner B. Intra-examiner correlation (comparison of the results of the two experiments using the guidance information by examiner A) and inter-examiner correlation (comparison of the results of the first experiment of examiner A with the results of the experiment of examiner B) were calculated with the kappa coefficients. The kappa coefficients were 1.0 for intra-examiner correlation and 0.798 for inter-examiner correlation. We confirmed that excellent agreement results were obtained for both intra- and inter-examiner correlations [48].

## Conclusions

In this paper, we proposed a machine learning-based semi-automatic classification method for breast lesions on CEUS called Ceusia-Breast. Experimental results on 119 cases showed a significant improvement in classification performance compared with conventional classification methods using only B-mode images. We also confirmed that the selected features are related to findings considered important in clinical practice. Furthermore, we verified the intra- and inter-examiner correlation in the guidance input for region extraction and confirmed that both correlations were in strong agreement.

The number of benign cases in this study was small compared to malignant cases, which is a limitation of this study because cases were collected at a second screening facility. Future work includes increasing the number of benign cases to confirm the effectiveness of our proposed method.

## Data Availability

The data that support the findings of this study are not openly available due to the limitation of data usage described in the document authorized by the ethics review board.

## Footnotes

1 In general, the area under the receiver operating characteristic curve is abbreviated as AUC. However, we use AUROC for the abbreviation because we have a different AUC (in terms of time intensity curve) in this paper

2 F1 score is the harmonic mean of precision and recall.

3 Specifically, it is an image obtained by extracting the fundamental component of echo signals from organs and contrast agent, and is imaged using a method different from conventional B-mode imaging, but it is referred to here as “B-mode” from the standpoint of its relevance to conventional methods.

4 In general, the area under the time intensity curve is abbreviated as AUC. However, we use AUTIC for the abbreviation because we have a different AUC (in terms of receiver operating characteristic curve) in this paper.
